# Impacts of kaolinite enrichment on biochar and hydrochar characterization, stability, toxicity, and maize germination and growth

**DOI:** 10.1038/s41598-024-51786-1

**Published:** 2024-01-13

**Authors:** Hamed A. Al-Swadi, Abdullah S. Al-Farraj, Mohammad I. Al-Wabel, Munir Ahmad, Adel R. A. Usman, Jahangir Ahmad, Mohammed Awad Mousa, Muhammad Imran Rafique

**Affiliations:** Soil Sciences Department, College of Food and Agricultural Sciences, King Saud University, P.O. Box 2460, 11451 Riyadh, Kingdom of Saudi Arabia

**Keywords:** Climate-change ecology, Ecology, Environmental sciences

## Abstract

In this study, biochar (BC) and hydrochar (HC) composites were synthesized with natural kaolinite clay and their properties, stability, carbon (C) sequestration potential, polycyclic aromatic hydrocarbons (PAHs) toxicity, and impacts on maize germination and growth were explored. Conocarpus waste was pretreated with 0%, 10%, and 20% kaolinite and pyrolyzed to produce BCs (BC, BCK10, and BCK20, respectively), while hydrothermalized to produce HCs (HC, HCK10, and HCK20, respectively). The synthesized materials were characterized using X-ray diffraction, scanning electron microscope analyses, Fourier transform infrared, thermogravimetric analysis, surface area, proximate analyses, and chemical analysis to investigate the distinction in physiochemical and structural characteristics. The BCs showed higher C contents (85.73–92.50%) as compared to HCs (58.81–61.11%). The BCs demonstrated a higher thermal stability, aromaticity, and C sequestration potential than HCs. Kaolinite enriched-BCs showed the highest cation exchange capacity than pristine BC (34.97% higher in BCK10 and 38.04% higher in BCK20 than pristine BC), while surface area was the highest in kaolinite composited HCs (202.8% higher in HCK10 and 190.2% higher in HCK20 than pristine HC). The recalcitrance index (R_50_) speculated a higher recalcitrance for BC, BCK10, and BCK20 (R_50_ > 0.7), minimal degradability for HCK10 and HCK20 (0.5 < R_50_ < 0.7), and higher degradability for biomass and HC (R_50_ < 0.5). Overall, increasing the kaolinite enrichment percentage significantly enhanced the thermal stability and C sequestration potential of charred materials, which may be attributed to changes in the structural arrangements. The ∑ total PAHs concentration in the synthesized materials were below the USEPA’s suggested limits, indicating their safe use as soil amendments. Germination indices reflected positive impacts of synthesized charred materials on maize germination and growth. Therefore, we propose that kaolinite-composited BCs and HCs could be considered as efficient and cost-effective soil amendments for improving plant growth.

## Introduction

Burning biomass, including wood, leaves, grass, and other materials, is likely to be a contributor to air pollution in many parts of the world^1^. The transition from a natural to an agricultural ecosystem resulted in the loss of 20–80 tons of carbon (C) per hectare, which had negative effects on the quality of water and soil as well as the productivity of biomass^2^. To maintain sustainable agriculture and environment, actions must be taken to reduce C losses from soil to atmosphere. Thermal carbonization is one of the suitable options for mitigation of climate change and minimize C losses. Pyrolysis and hydrothermal carbonization usually use high-temperature processes for transforming biomass into solid materials such as biochar (BC) and hydrochar (HC)^3–5^. The BC and HC have garnered global interest recently because of their considerable potential applications in various sectors, such as soil remediation, wastewater treatment, climate change mitigation, CO_2_ capture, and energy storage^6–10^.The C storage potential of BC and HC is a result of their high recalcitrance, which reduces the amount at which the photosynthetic processes of biomass fix C and release it into the atmosphere^11,12^. Also, aromatic ring structures in BC and HC make them more stable and stubborn material against thermal and microbial degradation^13,14^. Uncontrolled open burning of biomass releases harmful gases, enhancing environmental pollution. Therefore, converting biomass to BC can sequester C for 1000 years ^15^. On the other hand, HC can sequester C lower than that of BC^16^. It has been estimated that BC can sequester around 50% of the initial C^15^. Likewise, HC is also considered to be more stable than biomass^17^. The stability and recalcitrance of BC and HC are the most decisive factors that determine their C sequestration potential. Therefore, these properties of produced BC and HC depend on the type of feedstock, pyrolysis temperature, residence time, and pretreatment conditions^18^. The literature showed that the pyrolysis temperature, residence time, and heating rate are significantly correlated to pH, yield, surface area, fixed C, volatile matter, and ash content of biochar^18^. For instance, pyrolysis temperature is positively correlated with pH, surface area, microporous, and ash content, while having a negative relationship with yield, functional groups, and volatile matter ^18^. Additionally, BC and HC could improve the water holding capacity, fertility of soil, soil nutrients, and productivity of agriculture, while reducing the greenhouse gases emissions. Current researches have indicated that BC and HC can be employed as inexpensive alternative sorbents to eliminate a range of different organic and inorganic contaminants from environments.

Typically, BC is produced from organic waste materials such as agricultural waste, municipal sewage sludge, and manure, etc. under limited oxygen supply during pyrolytic processes^19–22^, while Pauline and Joseph^23^ mentioned that HC is produced by controlling the carbonization of biomass through thermochemical decomposition under pressure in hot compressed water at 180–300 °C for many hours. In terms of their physical and chemical characteristics, BC and HC (both chars) differ from one another, which makes them highly adaptable tools in a wide range of industries and environments. Despite these advantages, some disadvantages are associated with such materials. For instance, degradation of cellulose and lignin may generate phenolic chemicals such as PAHs and dioxins during the thermochemical decomposition of biomass, which could make the resultant material toxic and potentially pose a risk to the soil biota if applied as soil amendment^24,25^. Hence, to minimize the negative impacts of HC and BC on soil biota, it is crucial to examine the compositions of ecotoxicology of BC and HC before their application as soil amendments. Washing char materials with deionized water is a common practice to minimize toxicity potential^26^. Moreover, due to their surface characteristics and heterogeneous nature, BC and HC are not always successful^27^. Therefore, to enhance the physical and chemical characteristics of the charred materials, modification of the charred materials with other substances, such as acids, oxides, polymers etc. has been developed. Combining BC or HC with foreign materials may combine both materials' benefits, subsequently resulting in improved performance. However, in majority of cases, these materials could be either expensive, have a specific use^28^, could cause secondary pollution^29^, have a short half-life (ozonation) ^30^, or are complicated to prepare^31^. Therefore, scientists are looking for cheaper, greener, and more efficient materials to modify BC and HC for better results and broad applications. Clay minerals are extensively employed in agriculture, industrial engineering, and for the exploration, extraction, and refinement of fuel. Some of the essential physical and chemical characteristics which contribute to making clay minerals desirable and beneficial are size of particle, particle shape, surface chemistry, and surface area^32^. Kaolinite clay can assist as a potential candidate for this purpose due to its cost-effectiveness and abundance. In kaolinite clay minerals, one tetrahedral sheet is joined to one octahedral alumina sheet via oxygen atoms as layered silicate minerals^33^. Kaolinites have surface exchange sites, but no exchange sites exist between the layers^34^. Hence, using kaolinite clay minerals to modify the surface BC and HC could create new composites that can improve the performance of such composites for applications of environment^27,35,36^. Combining BC or HC with kaolinite may improve the porous structure of the composite due to better distribution of the kaolinite particles on BC or HC matrix^37^. On the other hand, kaolinite is abundant, cheap, environment-friendly, and chemically more stable. Thus, the kaolinite-composited BC and HC could have superior possibilities for applications as sorbents and amendments to soil^38–40^. The previous studies showed that biochar composites with kaolinite significantly enhanced the treated soil’s organic matter, ammonical nitrogen content (NH_4_-N), and cation exchange capacity (CEC) compared to the control^41^. In addition, the soil's NH_4_-N, organic matter, and CEC increased with an increase kaolinite percentage in the biochar composites^41^. Furthermore, the plant root, shoot length, and biomass significantly increased compared to the control^41^. Another study by Qiu et al.^42^ revealed that the lowest toxic Cd concentration and the highest stable Cd concentration were detected in the treated soil with kaolinite biochar composite. They also mentioned that the kaolinite also enhanced the stability of biochar. Therefore, we hypothesized that compositing biochar/hydrochar with kaolinite clay minerals might combine both materials' benefits, consequently resulting in a stable and applicable material for environmental and agricultural applications.

To the best of our knowledge, very limited researches have been attempted to combine and compare the composites of BC and HC with kaolinite. Therefore, the main aims of this research were to: (1) synthesize low-cost composite materials of BC and HC with kaolinite natural deposits via pyrolysis and hydrothermal carbonization, (2) characterize the synthesized materials for chemical, proximate, elemental, and structural properties, and evaluate stability, and C sequestration potential, and (3) explore the potential toxicity of the synthesized materials by analyzing PAHs compounds and investigating impacts on maize (*Zea mays* L*.*) seed germination.

## Materials and methods

### Synthesis of materials

#### Synthesis of kaolinite-biochar composite

Conocarpus waste was gathered from the King Saud University Campus in Riyadh, Saudi Arabia. The conocarpus waste was then washed, air-dried, ground, passed through a 1000 μm sieve, and termed as BM. Kaolinite deposits were obtained from the Al-Zobaira region in the Hael governorate (N: 27.916207, E: 43.711223). Kaolinite deposits were dried, ground, and washed with warm deionized water to remove gypsum. Later on, the kaolinite deposits were washed with deionized water and shaken many times for removal of soluble salts. Thereafter, the kaolinite deposits were dried at 105 °C for 4 h, and ground by grinder (Rotary Cup Mill (BICO), Sepor Company, India) to less than 100 μm size. The suspension of kaolinite was made by adding 0, 1, and 2 g of kaolinite powder to 500 mL of deionized water; the mixture was ultrasonically sonicated for 30 min with a frequency of 50 kHz (Ultrasonic sonicator Q700, Qsonica, Newtown, CT, USA). Subsequently, conocarpus BM (10 g) was immersed into the kaolinite suspension and stirred for 1 h. Following separation from the mixture, the kaolinite-BM was dried in an oven at 80 °C. Then, the kaolinite-BM was placed in a tightly sealed stainless-steel container (length: 22 cm and diameter: 7 cm), put in the muffle furnace (WiseTherm; Daihan Scientific, Gangwon-do, South Korea), and pyrolyzed at 600°C for 1 h under limited oxygen supply. The untreated feedstock was also used to prepare BC without kaolinite modification (i.e., pristine BC) with the same conditions of pyrolysis in the furnace. The pristine BC and kaolinite-BC composites were cooled and washed with deionized water many times for impurities removal, then dried in an oven, ground, sieved through a 100 μm screen, and stored in a container for further analyses. The synthesized pristine BC and kaolinite-modified BC composites with 10% and 20% kaolinite were henceforth referred to as BC, BCK10, and BCK20, respectively.

#### Synthesis of kaolinite-hydrochar composite

The dried kaolinite-BM mixture was used for the preparation of kaolinite-HC composite. 60 g of each kaolinite-BM mixture was added to 600 mL (1:10 v/v) DI water, stirred for 1 h, then placed in a tightly sealed stainless-steel container (length: 30 cm and diameter: 7 cm) and put in the muffle furnace (WiseTherm; Daihan Scientific, Gangwon-do, South Korea) at 200 °C for 6 h, then allowed to cool. The untreated feedstock was also used to prepare HC without kaolinite modification (i.e., pristine HC). After oven-drying, the pristine HC and kaolinite-HC composites were washed with deionized water many times for impurities removal, dried in an oven, ground in a mortar, sieved to pass 100 μm and stored in a container for further analyses. The synthesized pristine HC and kaolinite-modified HC composites with 10% and 20% of kaolinite were henceforth referred to as HC, HCK10, and HCK20, respectively.

### Characterization of the synthesized materials

All synthesized materials, such as BM, BCs (BC, BCK10, and BCK20), and HCs (HC, HCK10, and HCK20) were conducted to various chemical, proximate, and ultimate analyses.

#### Yield, proximate and chemical analyses

Equation (1) was applied to estimate the yield percentage of the synthesized materials:

1 $$Yield \left(\%\right)=\frac{Weight\,of\,Biomass-Weight\,of\,BC\,or\,HC\,composite}{Weight\,of\,Biomass} \times 100$$

The proximate analysis of synthesized materials was carried out to measure the percentage of moisture content, ash, volatile matter, and fixed carbon^43,44^. The pH of the synthesized materials was measured at a 1:25 solid/water ratio^45^; EC was determined in the same ratio extraction. The method of ammonium acetate extraction was conducted to determine the CEC values of the synthesized materials^46^.

#### Ultimate analysis

The CHNS analyzer (PerkinElmer series II, Walttham, USA) was used for the ultimate analysis (indicated by elemental composition) of the synthesized materials to measure C, nitrogen (N), hydrogen (H), and sulfur (S). Equation (2) was used to calculate the percentage of oxygen (O) in the synthesized materials:

2 $$ O~\left( \%  \right) = ~100~{-}~\left( {{\text{C}}~ + {\text{H}}~ + {\text{N}} + {\text{S}}~ + ~{\text{ash}}\% } \right)~  $$

According to the obtained results of elemental composition, the aromaticity and polarity index of the synthesized materials were also calculated using the elemental molar ratios of O/C and H/C.

#### XRD, SEM, FTIR, BET, TGA, zeta potential and hydrodynamic size analyses

X-ray diffraction (XRD) (MAXima X XRD-7000, Shimadzu, Japan) was employed to observe the mineralogical composition of the synthesized materials. The surface morphology and structural changes of synthesized materials were analyzed by capturing images on scanning electron microscopy (SEM) (SEM, EFI S50 Inspect, Netherlands). The aluminum stubs coated with adhesive carbon tape (12 mm; PELCO, UK) were used to spread the samples and then coated for 60 s with nano-gold particles using a 108 Auto/SE Sputter Coater (Ted Pella Inc. USA). The images were captured in a high vacuum at an acceleration voltage of 30 kV and a magnification of 3000. To analyze the functional groups of the synthesized materials, a Fourier Transform Infrared Spectrometer (FTIR, Bruker Alpha-Eco ATR-FTIR, Bruker Optics Inc) was employed. The Brunauer-Emmett-Taller (BET) method using a surface area and porosity analyzer (TriStar II 3020, Micromeritics, USA) was used to analyze the surface area, total pore volume, and pore size. Thermogravimetric analysis (TGA) was performed to display the weight loss of the synthesized materials (DTG-60H, Shimadzu, Japan). With temperature increases from 25 to 1000 °C, weight loss of the synthesized materials was observed. The zeta potential of the synthesized materials was measured using the techniques of dynamic light scattering by determining the electrophoretic mobility of 1 g L^−1^ for the various particle suspensions with Zetasizer (Zetasizer Nano ZS, Malvern, UK). Using Laser Doppler Velocimetery (Zetasizer Nano ZS, Malvern, UK), the average hydrodynamic size of the synthesized material particles was determined in aqueous suspensions.

#### Estimation of thermal stability

Harvey et al.^47^ established the recalcitrance index (R_50_), which they calculated using moisture and ash free TGA analytical data to quantify the materials' relative thermal degradability by following Eq. (3):

3 $${R}_{50}= {T}_{50,x}/{T}_{50,graphite}$$

where *T*_*50,x*_ and* T*_*50,graphite*_ are the temperatures of the moisture and ash corrected TGA thermograms of the synthesized materials and graphite, respectively (weight loss because of oxidation of C only), of which 50% of the weight is lost by oxidation or volatilization.

According to Harvey et al.^47^, Eq. (4) was used to correct the TGA thermograms for contents of moisture and ash:

4 $$  W_{{i,cor}}  = 100 + [100 \times \left( {W_{{i,uncor}} {-}W_{{200,uncor}} } \right]/\left( {W_{{200,uncor}} {-}W_{{cutoff,uncor}} } \right)  $$

where *W*_*i,cor*_ and *W*_*i,uncor*_ represent, respectively, the corrected and uncorrected percent weight loss of the initial material, while *W*_*200,uncor*_ represents the initial material's weight loss in percentage up to 200 °C (which corresponds to water loss in the material). *W*_*cutoff,uncor*_ is the weight loss at the temperature where no more oxidation occurred.

Materials can be categorized into three groups based on R_50_ values^47^:

- R_50_ ≥ 0.7 = highly recalcitrant
- 0.7 ˃ R_50_ ≥ 0.5 = minimally degradable
- R_50_ < 0.5 = highly degradable

Equation (5) provided by Zhao et al.^48^ was used to calculate the percent carbon sequestration potential (CS).

5 $$\mathrm{CS }(\mathrm{\%})=\frac{Yield\, \left(\%\right)\, x \,C\%\, material \,x\, R50}{C\%\, feedstock}$$

where C %_material_ and C %_feedstock_ are the percent carbon content of the material and feedstock, respectively.

#### PAHs analysis

The special extraction method provided by El-Saeid et al.^49^ was used by adding 1 g of material and 1 mL of DI water to a 50 mL centrifuge tube, after a short vortex, the mixture was allowed to homogenize for roughly 10 min. Each material received 6 mL of acetonitrile, and the sample was shaken for 5 min. Individual materials were placed in the centrifuge tube, and the Mylar pouch's citrate salts (ECQUEU750CT-MP) were added. Then, materials were shaken immediately for at least 2 min, followed by a 5 min and then centrifugation at ≥ 3500 rcf. The cleanup of materials was executed by transporting a 1.5 mL aliquot of supernatant to a 2 mL CUMPSC18CT (MgSO_4_, PSA, C18) dSPE tube. Next, vortexing of materials was carried out for 2 min and then centrifuged for 2 min at high rcf (i.e., ≥ 5000). The supernatant solution was immediately transferred into a 1.8 mL GC vial using a 0.2 µm syringe filter. Finally, PAHs in the solution were estimated by GC–MS/MSTQD.

The EPA method was applied to adjusted the GC MS/MS conditions (SVOC 8270). For the procedure development of GC MS/MS, the auto SRM was carried out. The improved procedure was divided into quantifier and qualifier ions to acquire good sensitivity. With a maximum of 51 transitions per segment, scanning was successfully completed in each segment (500–700 MS). Prior to each batch of analysis, MS was automatically tuned, while nitrogen and argon were employed as collision gases, and helium gas was employed as a carrier gas from a registered Linde gas (SiGas, Saudi Arabia). A total of 17 PAHs were analyzed from all synthesized materials.

### Germination test

This study complies with national and international regulation and legislation for the maize (*Zea mays* L.) plant. The methods involved in this study are in accordance with the IUCN Policy Statement on Research Involving Species at Risk of Extinction and the Convention on the Trade in Endangered Species of Wild Fauna and Flora. The International Biochar Initiative recommends a quick and easy germination inhibition analysis to determine whether there are any unfavorable substances in the synthesized materials. A germination experiment was conducted by growing plants in Petri plates to measure the toxic impacts of the synthesized materials. Briefly, filter papers (Whatman 42) were used to germinate maize seeds. The phototoxicity of the synthesized materials was assessed by comparing their germination results with those of the control (without any amendment). Briefly, the Petri plate size was (90 mm × 12mm) and the filter papers were cut to fit the Petri plate. After that, the Petri plates were soaked in 5 mL of DI water. Then, 0.2 and 0.4 g of pristine BC, HC, and kaolinite-synthesized BC and HC (BCK10, BCK20, HCK10, and HCK20) were sprayed separately on the particular filter paper. Next, 10 maize seeds were added on each filter paper. Subsequently, the Petri plates were covered and put in the darkness for 48 h at 25°C. Then, for the next 11 days, there were cycles of 16 h light and 8 h darkness. The number of seeds that germinated in each treatment was used to calculate the percentage of germination rate^8^. Moreover, the fresh and dry weights of maize seedlings and shoot and root length were also measured. Triplicates of each treatment were carried out.

Equation (6) of the germination index (GI) is as follows:

6 $$GI (\%)=\frac{no\, of\, germinated\, seeds}{no\, of\, total\, seeds} \times 100$$

### Statistical analysis

Using the Statistics 8.1 program, the obtained data were statistically analyzed^50^. Descriptive statistics were used to calculate the average and standard deviation. The least significant difference (LSD) test was applied to compare the treatment means, with a significant level of 0.05.

## Results and discussion

### Characterization

#### Proximate and chemical analyses

The results of the chemical and proximate analyses of the fabricated materials are presented in Table 1. The results revealed that the HC materials (HC, HCK10, and HCK20) had a higher yield than the BC materials (BC, BCK10, and BCK20). In addition, the highest yield was found in HCK20, while the lowest yield was found in BC. On the other hand, increased yield was associated with an increasing percentage of kaolinite, which was noticed in BCK20 and HCK20 compared to pristine. The yield order was in the order: HCK20 (64.35%) ˃ HCK10 (62.19%) ˃ HC (56.74%) ˃ BCK20 (36.84%) ˃ BCK10 (32.89%) ˃ BC (24.15%). Therefore, the HC materials showed a higher yield than the BC materials. The lower yield of pristine BC materials can be attributed to the higher volatile matter loss and a higher weight loss from biomass through pyrolysis than those of HC materials, which were made through hydrothermal carbonization in an airtight container. Hence, the higher yield of kaolinite-synthesized materials indicated more thermal stability than pristine. The highest yield of kaolinite-synthesized materials could be related to the highly resistant nature of kaolinite against thermal degradation^42^. The volatiles decreased with an increasing percentage of kaolinite and carbonization. This reduction was 3.94 times for BC, 4.13 times for BCK10, and 4.07 times for BCK20 than that of BM, while 1.44 times for HC, 1.67 times for HCK10, and 2.02 times for HCK20 than that of BM. Likewise, the fixed C was higher in pristine BC and HC as compared to BC and HC-based materials. The highest fixed C was observed in BC (70.20%), while the lowest was in BCK20 (25.59%) among the synthesized materials. In all synthesized materials, BC materials were more recalcitrant than HC materials^8^. The highest ash contents were found in BCK20, while the lowest were in HC. The ash contents increased with increased kaolinite percentage in synthesized BC and HC materials. The ash contents were increased by 2.43, 12.87, 18.15, 0.91, 6.35, and 9.46-folds in BC, BCK10, BCK20, HC, HCK10, and HCK20, respectively, as compared to BM, indicating the formation/condensation of the compounds of minerals in these materials through pyrolysis^51^. In comparison to HC-based materials, the increased ash contents in BC-based materials were related to the thermal oxidation of organic compounds during the pyrolysis process^8^. The BM displayed a higher moisture content of 2.73%, while it was higher in HCs (HC = 2.41%, HCK10 = 2.11%) and HCK20 = 1.46%) as compared to BCs (BC = 0.87%, BCK10 = 1.02%, and BCK20 = 0.41%). The HC materials had a higher moisture content due to being hydrothermally pretreated.

**Table 1:** Chemical and proximate analyses of the biomass and synthesized materials.

Material	Moisture (%)	Volatiles (%)	Fixed carbon (%)	Ash (%)	pH (1:25)	EC (dSm^−1^)	Yield (%)	CEC (cmol kg^−1^)
BM	2.73	86.22	7.13	2.91	5.31	0.98	–	–
BC	0.87	21.86	70.20	7.07	8.72	0.44	24.15	16.3
BCK10	1.02	20.87	40.67	37.45	9.35	0.41	32.89	22.0
BCK20	0.41	21.18	25.59	52.82	10.97	0.66	36.84	22.5
HC	2.41	59.67	35.27	2.65	6.22	0.25	56.74	132.8
HCK10	2.11	51.60	27.80	18.48	5.22	0.31	62.19	123.0
HCK20	1.46	42.77	28.24	27.53	4.48	0.31	64.35	124.8

Biochar (BC), BC with 10% kaolinite enrichment (BCK10), BC with 20% kaolinite enrichment (BCK20), Hydrochar (HC), HC with 10% kaolinite enrichment (HCK10), HC with 20% kaolinite enrichment (HCK20), BM: biomass.

The finding displayed that the pH of BC materials elevated with an increasing kaolinite percentage during pyrolysis. In contrast, the pH of HC materials decreased with an increasing kaolinite percentage during hydrothermal carbonization. The highest pH value was 10.97 in BCK20, while the lowest was 4.48 in HCK20. The pH increased by 3.14, 4 and 5.7 units in BC, BCK10 and BCK20, respectively, compared with the BM, indicating the elimination of acidic functional groups and concentration of basic functional groups^52^. Furthermore, with increasing the pyrolysis temperature, the recalcitrant cationic species (Ca^+2^, Mg^+2^, Na^+^) condensed in BC materials, which could also cause increased pH^53,54^. On the other hand, the pH values of the HCK10 and HCK20 were decreased by only 0.09 and 0.83 units, respectively, as compared with BM, indicating the minimal removal of basic functional groups^52^. The pH value of the HC increased by 0.91 units compared with the BM. Likewise, the EC of all materials decreased with pyrolysis and hydrothermal carbonization, which could be due to washing all the materials before analysis to remove surface basicity^55^, as well as the dissolved salts released into the liquid phase during hydrothermal carbonization^56^. The highest cation exchange capacity (CEC) was shown in HC (132.8 cmol kg^−1^), while the lowest CEC was in BC (16.3 cmol kg^−1^). With pyrolysis, the CEC was increased by 34.97% for BCK10 and 38.04% for BCK20 as compared to pristine BC, which is attributed to an increase in surface functional groups^57^. Contrarily, with hydrothermal carbonization, the CEC was decreased by 7.38% for HCK10 and 6.02% for HCK20 compared with HC. Nevertheless, the CEC for HC materials was several times higher than that for BC materials due to more oxygen-containing functional groups on the surface of HC materials^58–61^.

#### X-ray diffraction, SEM, and FTIR analyses

**Figure 1:**
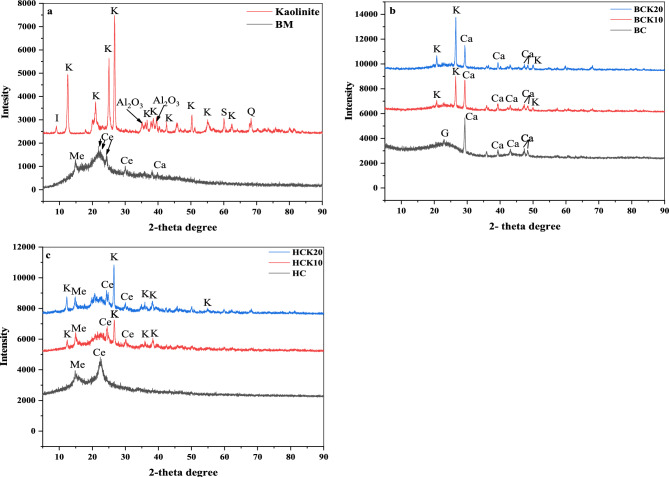
X-ray diffraction of (**a**) kaolinite and BM, (**b**) BC materials, and (**c**) HC materials. (*Biochar (BC), BC with 10% kaolinite enrichment (BCK10), BC with 20% kaolinite enrichment (BCK20), Hydrochar (HC), HC with 10% kaolinite enrichment (HCK10), HC with 20% kaolinite enrichment (HCK20), BM: biomassBC*: biochar, *BCK10*: biochar composite with 10% of kaolinite, *BCK20*: biochar composite with 20% of kaolinite, *HC*: hydrochar, *HCK10*: hydrochar composite with 10% of kaolinite, *HCK20*: hydrochar composite with 20% of kaolinite, *BM*: biomass, *K*: kaolinite, *Me*: mellite, *Ce*: cellulose, *S*: saponite, *Ca*: calcite, *G*: graphite, *Q*: quartz).

The XRD spectra of the BM, kaolinite deposits, and synthesized materials are shown in Fig. 1. The various visible peaks on the spectra of all the synthesized materials demonstrate the presence of crystalline minerals and inorganic materials. The XRD patterns of raw materials, such as kaolinite clay deposits and BM, were identified. In the XRD pattern of kaolinite deposits, the strongest intense peaks of kaolinite in XRD pattern of kaolinite deposits were identified at 2θ = 12.46°; 25.06° and 26.68° (Fig. 1a)^62–64^ and lower diffraction kaolinite peaks than previous peaks were revealed at 2θ = 36.7°; 39.50°; 42.56°; 50.3°; 55.16° and 62.4°^65–70^. Four peaks of cellulose in BM are displayed at = 21.8; 22.4°; 24.3° and 30°, peak of carbon-containing minerals mellite is at 2θ = 14.8°, and calcite at 2θ = 39.78° (Fig. 1a)^71–74^. The kaolinite peaks were found in BC-based materials and HC-based materials, which endorsed successfully implanted kaolinite onto the BC and HC matrix. Peak shifting is a sign of interactions between kaolinite and BC or HC during synthesizing the composite. Peaks of kaolinite at 2θ = 20.7°; 26.68°; 39.5° and 50.3° in BCK10 and BCK20 were shifted to 20.78°; 26.6°; 39.26° and 50°, respectively, and 2θ = 25.06° in BCK10 was shifted to 25.2°, and 2θ = 36.86° in BCK20 was shifted to 36.48° (Fig. 1b)^64,66,68,75^. Similarly, peaks of kaolinite at 2θ = 12.46°; 26.68°; 36.1°; 38° and 42.56° in HCK10 and HCK20 were shifted to 12.26°; 26.62°; 36.14°; 38.32° and 42.46°, respectively, and 2θ = 39.5° in HCK10 was shifted to 39.44°, and 2θ = 55.1° was shifted to 54.78° (Fig. 1b)^64,67,76,77^. The other peaks in BC-based materials were impurities corresponding to calcite and quartz; and cellulose and mellite for HC-based materials. The XRD analysis of BC (Fig. 1b) displayed peaks at 2θ = 23.04°, which indicated the presence of graphite^78^. Other peaks were identified at 2θ = 29.3°; 39.44°; 43.1°; 47.34° and 48.28°, and indicated the presence of calcite^52,79,80^. Likewise, the peak of cellulose in HC is at 2θ = 22.4°^72^ and the peak of mellite is at 2θ = 14.8° (Fig. 1c). In BC-based materials, mellite was lost during the pyrolysis process of BM, while it still exists with HCs. Therefore, the changes in the results of the kaolinite-synthesized BC and kaolinite-synthesized HC showed that the synthesis method used successfully implanted kaolinite onto the BC and HC matrix.

**Figure 2:**
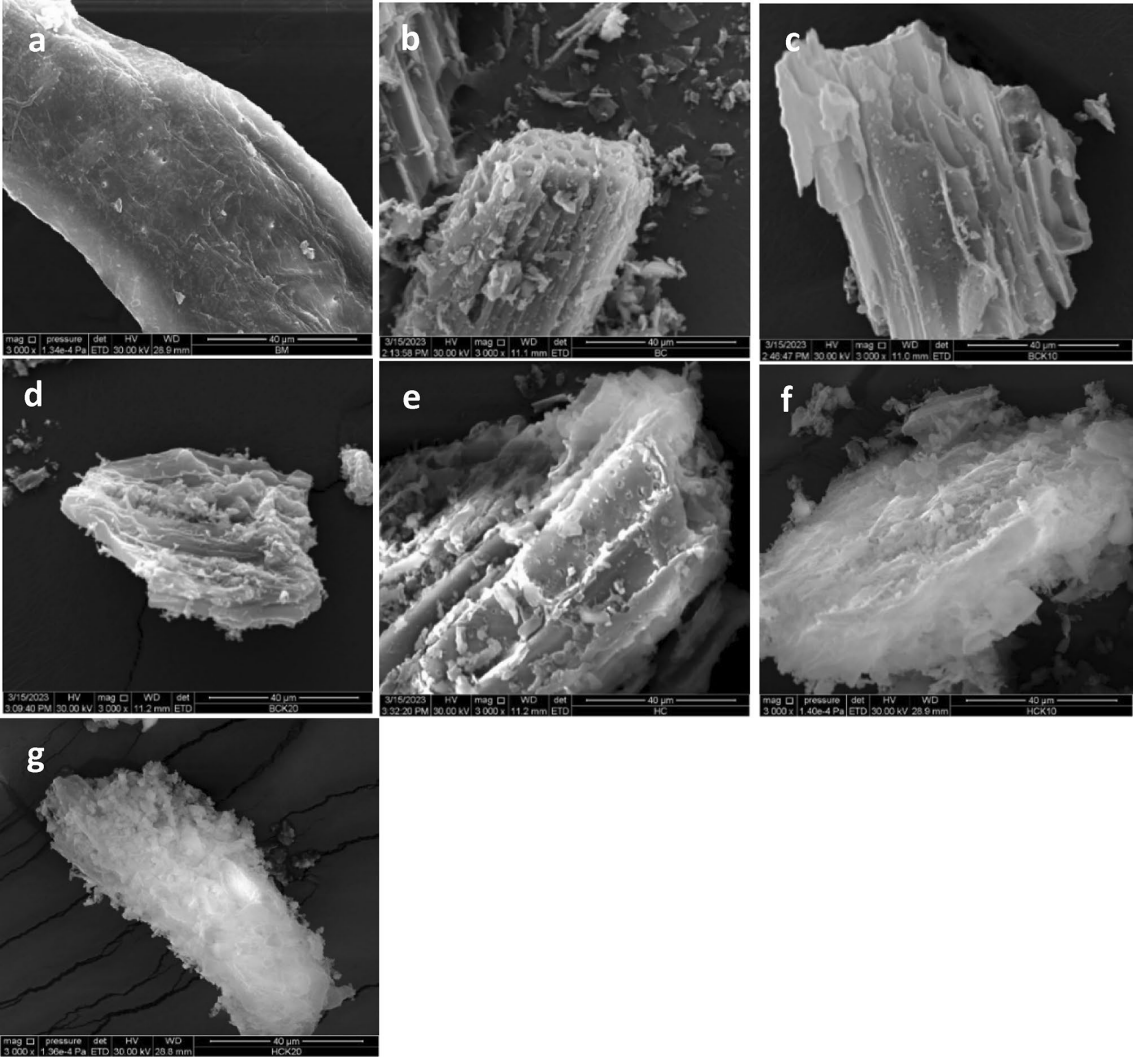
Scanning electron microscopy images of (**a**) BM (**b**) BC, (**c**) BCK10, (**d**) BCK20, (**e**) HC, (**f**) HCK10 and (**g**) HCK20. (Biochar (BC), BC with 10% kaolinite enrichment (BCK10), BC with 20% kaolinite enrichment (BCK20), Hydrochar (HC), HC with 10% kaolinite enrichment (HCK10), HC with 20% kaolinite enrichment (HCK20), BM: biomass).

SEM images in Fig. 2 depict the surface morphology of the synthesized materials. SEM images are highly useful to obtain minute details about the structure of synthesized materials and their modifications. In addition, comparing pristine BC and HC with their modified and raw materials would therefore allow us to make judgments on morphological changes during pyrolysis and hydrothermal carbonization. Pyrolysis and hydrothermal carbonization converted the crystalline surface of BM (Fig. 2a) into porous and amorphous materials, as presented in Fig. 2b–g. The surface area of the BC-based materials and HC-based materials was generally coated by thin film structures and were more irregular on the BC and HC surfaces (Fig. 2c–g), indicating that after zooming in at 3000 × magnification, the kaolinite well onto the surfaces and within the pores of BC and HC^81^. The decomposition and volatilization of biomass caused a small number of pores with different sizes to appear in pristine BC and HC. In addition, the SEM images showed that kaolinite was not entirely covering the surfaces of the BC-based materials^82,83^, while kaolinite was entirely covering the surfaces of the HC-based materials.

**Figure 3:**
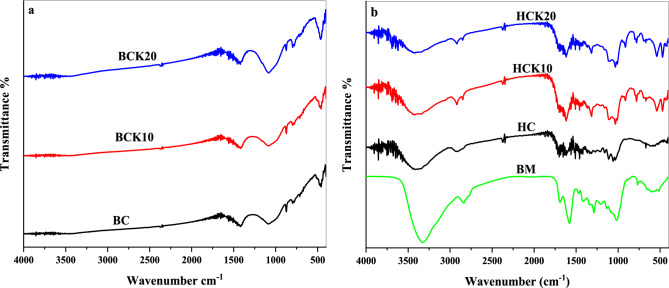
Fourier Transformation Infrared (FTIR) analysis (**a**) BC materials and (**b**) HC materials and BM (Biochar (BC), BC with 10% kaolinite enrichment (BCK10), BC with 20% kaolinite enrichment (BCK20), Hydrochar (HC), HC with 10% kaolinite enrichment (HCK10), HC with 20% kaolinite enrichment (HCK20), BM: biomass).

According to Li et al.^84^, the surface functional groups, particularly O-containing functional groups, may facilitate the chemical adsorption of BC and HC. Figure 3 displays the FTIR spectra of synthesized materials in the range of 400–4400 cm^−1^. A broad band has been explored at 3300–3800 cm^−1^ in BM, representing the presence of O–H bonding^85^, which continued to appear during hydrothermal carbonization and vanished during pyrolysis. The structural–functional groups found in the BC and HC materials included C=C, O–H, C–O, C=O, C–H, Si–O–Al, Si–O–Si, Si–O, N–H, C–OH, CH_2_ and C–N. It is evident that some functional groups, such as C–H and C=O (between 1413 and 1462 cm^−1^) were shared by BC and HC materials^86^, C–O and O–H (1033 cm^-1^)^87^. For BC-based materials, some peaks appeared with increasing kaolinite, such as; Si–O–Si groups at 465 and 469 cm^−1^^88^, Si–O at 791 cm^−1^^89^ and C–O, C–N at 1083 cm^−1^^90^^,^^91^. Therefore, those peaks indicated that kaolinite was successfully loaded onto the BC matrix^92^. The HC materials showed more bands than BC materials, which could be due to minimal losses of functional groups. Likewise, some peaks appeared with increasing kaolinite, such as Si–O–Si at 469 cm^−1^^85^, Si–O and Al–O vibrations at 762, 696 and 539 cm^−1^^93^, Si–O at 784 cm^−1^^94^. On the other hand, the same peaks appeared in HC, HCK10, and HCK20 composites, such as; C–O and O–H at 1033 cm^−1^^87^, C–O–C at 1111 cm^−1^^95^, CH_3_ at 1440 cm^−1^^96^, C=O at 1510 cm^−1^, C=O at 1510 cm^−1^^97^, COOH at 1700 cm^−1^^98^, C–H at 2921 cm^−1^^99^, and O–H (between 3300 and 3800 cm^−1^) ^82^. Moreover, some bands were not found in BC and HC alone, but when composited with kaolinite, such bands were visible. Therefore, this finding can further assist in predicting the impact of kaolinite-synthesized BCs and HCs on the removal efficiency of these composites for various pollutants.

#### Size and surface characteristics

The BET surface area, pore size, and pore volume of the BM and synthesized materials are shown in Table 2. BC materials have a higher surface area than HC materials. The highest surface areas were found in BC (290.89 m^2^ g^−1^), and the lowest in HC (5.32 m^2^ g^−1^). Moreover, it was detected that the surface areas of the BCK10 and BCK20 (225.14 and 180.40 m^2^ g^−1^) were reduced compared to the pristine BC (290.89 m^2^ g^−1^), indicating that the pores on the BC might have been covered/clogged by kaolinite^39^. Conversely, the pristine HC suffered agglomeration and revealed a lower surface area^97,100^. Therefore, the surface area of the HCK10 and HCK20 was higher than that of HC. The surface area of the HCK10 and HCK20 was 16.11 and 15.44 m^2^ g^−1^, which was threefold higher than the pristine HC (5.32 m^2^ g^−1^). The composite interposes retained the kaolinite particles, increasing the surface area of kaolinite-HC^43,101^. Contrarily, the BCK10 and BCK20 pore sizes were 38.31 and 28.17 Å, which were higher as compared to BC (28.81Å). On the contrary, the pore sizes of HCK10 and HCK20 were 145.90 and 175.92 Å, which were lower as compared to HC (187.71 Å). The highest pore sizes was appeared in HC (187.71 Å), while the lowest was in BCK20 (28.17 Å). However, with increasing the amount of kaolinite deposit, the surface area decreased by 22.68% in BCK10 and 38% in BCK20 compared with pristine BC, while kaolinite addition increased the surface area of HCK10 by 203% and HCK20 by 190% as compared to pristine HC. Overall, the pore size of the HC materials was more than 10 times larger than that of the BC materials. The previous studies showed that the low surface area of zeolite and silica-composited BC is due to plugging of pores in the existence of minerals^51^. Yao et al.^35^ also mentioned that the blockage of BC pores by clay minerals particles could be the cause of the decreased surface area of clay biochar composites. The hydrodynamic size of the particles of the synthesized materials in aqueous suspensions was determined (Table 2). Any particle aggregate or particle with an equivalent diameter is seen by Dynamic Light Scattering (DLS) to reflect a similar size. The average size of particles (hydrodynamic size) was 2.63 μm for BC, 2.34 μm for BCK10, 2.73 μm for BCK20, 2.19 μm for HC, 2.38 μm for HCK10, and 3.10 μm for HCK20. These results of particle size analyses suggested that HC showed the minimum particle size, while HCK20 showed the maximum particle size.

**Table 2:** Surface area, pore volume, pore size, hydrodynamics size and zeta potential analyses result of BC and HC materials.

Material	Surface area (m^2^ g^−1^)	Pore volume (cm^3^ g^−1^)	Pore size (Å)	Hydrodynamics size (µm)	Zeta potential (mV)
BC	290.89	0.209	28.81	2.63	− 25.06
BCK10	225.14	0.216	38.31	2.34	− 25.08
BCK20	180.40	0.127	28.17	2.73	− 25.06
HC	5.32	0.025	187.71	2.19	− 24.98
HCK10	16.11	0.059	145.90	2.38	− 24.66
HCK20	15.44	0.068	175.92	3.10	− 21.68

Biochar (BC), BC with 10% kaolinite enrichment (BCK10), BC with 20% kaolinite enrichment (BCK20), Hydrochar (HC), HC with 10% kaolinite enrichment (HCK10), HC with 20% kaolinite enrichment (HCK20).

Colloidal dispersions have an electrokinetic potential known as zeta potential, and the value of this potential is influenced by the surface charge of the individual particles. In our study, the zeta potential values of the synthesized materials were determined as a function of the solution pH (Table 2). The zeta potential of the pristine BC and BC-based materials ranged from − 25.06 to − 25.08 mV; however, the zeta potential of the pristine HC and HC-based materials ranged from − 21.68 to − 24.98 mV. The highest zeta potential was shown in BCK10 (− 25.08 mV), while the lowest was in HCK20 (− 21.86 mV). Consequently, the negative charge of pristine BC and BC-based materials is slightly higher than that of pristine HC and HC-based materials. Hence, the zeta potential of all the synthesized materials was negative, indicating that all surfaces of the synthesized materials are negatively charged. Therefore, a larger zeta potential means a larger negative charge, which is beneficial for remediation, especially with removing cationic ions.

#### Elemental composition and carbon stability

The elemental composition of the synthesized materials is presented in Table 3. In comparison to BM, thermal treatment enhanced the total C contents of BC and HC materials. An increased degree of carbonization may be the cause of rising C contents with pyrolysis and hydrothermal carbonization. The higher contents of C were observed in BC, and the lowest was observed in HCK20 among the synthesized materials. HC materials had higher H contents ranging from 5.33 to 5.54%, while BC materials ranged from 1.08 to 1.41%. Among the synthesized materials, the maximum N contents were in BCK20, while the minimum was in BC. However, the C contents of the BC, BCK10, BCK20, HC, HCK10, and HCK20 were increased by 47.8%, 44.1%, 43.6%, 18.3, 20.9%, and 17.8%, respectively, as compared to BM. The C content was higher in pristine BC, which decreased with kaolinite modification in BC-based materials, such as 6.59% in BCK10 and 7.32% in BCK20. Meanwhile, the C content was increased in HCK10 (3.37%) and slightly decreased in HCK20 (0.52%) compared to HC. The increasing lignin content in biomass has been mentioned to enhance carbonization and increase the content of C in BC^102,103,105^. Other studies reported that cellulose and hemicelluloses also significantly affect on the C content^104,105^. On the other hand, a reduction of H contents in BC materials was more than that of HC materials. Compared to BM, the reduction of H contents for BC, BCK10, BCK20, HC, HCK10, and HCK20 was 76.3%, 78.4%, 82.0%, 10.8%, 7.3%, and 9.9%, respectively. According to previous studies, Gai et al. ^106^ reported that the decrease in H content of BC was due to the loss of water, gaseous H_2_, hydrocarbons, and tarry vapors. The total N contents decreased with pyrolysis and hydrothermal carbonization in BC (27.3%), HC (3.1%), and HCK10 (11.9%), while it increased in BCK10 (21.5%), BCK20 (32.6%), and HCK20 (18.9%) compared to BM. Furthermore, the contents of N increased with increased kaolinite modification of BC and HC, which were higher in BCK20 and HCK20. The total O contents decreased with pyrolysis and hydrothermal carbonization of biomass in all materials. The reduction in O was observed in BC (97.1%), BCK10 (92.5%), BCK20 (94.5%), HC (27.1%), HCK10 (30.8%), and HCK20 (29.6%), compared to BM. Moreover, BC-based materials showed higher O contents compared to pristine BC, while HC-based materials slightly presented lower O contents compared to pristine HC. Dehydration, volatilization, and depolymerization could be the causes of the decrease in O contents with hydrothermal carbonization and pyrolysis^107^.

**Table 3:** Elemental composition, their molar ratios, recalcitrant index (R_50_), temperature corresponding to 50% weight loss of water and ash-free composites (T_50_), and carbon sequestration potential (CS) of the synthesized materials.

Material	N (%)	C (%)	H (%)	S (%)	O (%)	O/C	H/C	T_50_	R_50_	CS (%)
BC	4.96	92.50	1.41	0.00	1.13	0.009	0.182	690.33	0.78	46.43
BCK10	8.68	86.40	1.29	0.73	2.90	0.025	0.178	714	0.81	47.63
BCK20	10.11	85.73	1.08	0.96	2.13	0.019	0.150	697.67	0.79	51.63
HC	6.61	59.12	5.33	0.58	28.35	0.360	1.075	374	0.42	29.16
HCK10	6.01	61.11	5.54	0.43	26.91	0.331	1.080	445.33	0.50	39.32
HCK20	8.41	58.81	5.39	0.00	27.39	0.350	1.092	447.67	0.51	39.94
BM	6.82	48.32	5.98	0.00	38.88	0.604	1.474	354.33	0.40	–

Biochar (BC), BC with 10% kaolinite enrichment (BCK10), BC with 20% kaolinite enrichment (BCK20), Hydrochar (HC), HC with 10% kaolinite enrichment (HCK10), HC with 20% kaolinite enrichment (HCK20), BM: biomass.

**Figure 4:**
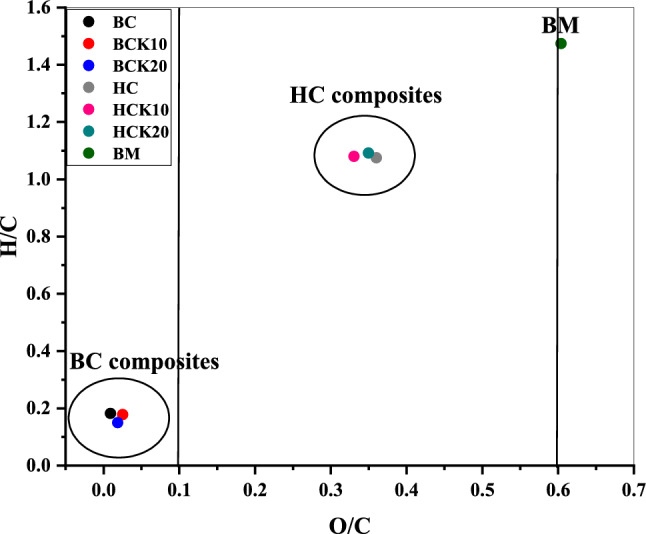
Van Krevelen diagram of elemental ratios of H/C and O/C of synthesized materials. (Biochar (BC), BC with 10% kaolinite enrichment (BCK10), BC with 20% kaolinite enrichment (BCK20), Hydrochar (HC), HC with 10% kaolinite enrichment (HCK10), HC with 20% kaolinite enrichment (HCK20), BM: biomass).

Figure 4 illustrates the Van Krevelen diagram, frequently used to calculate the molar O/C and H/C ratios to compute the BC and HC material’s recalcitrance. With pyrolysis and thermal carbonization, conocarpus waste biomass is dehydrated and depolymerized to produce smaller dissociation products^24,108^. Reduced H/C and O/C molar ratios showed reduced polarity and a greater degree of aromaticity, which in turn increased the stability of BC^55^. Also, another research mentioned that the higher stability of BC composites is attributed to greater polyaromatic carbon content compared to HC composites^109^. From Table 3 and Fig. 4, the surface polarity index (i.e., indicated by O/C molar ratio) of BC and HC materials decreased with pyrolysis and hydrothermal carbonization compared with BM, indicating a decrease in the hydrophobicity of these materials. Overall, the highest values of O/C and H/C (0.604 and 1.47) were found for the BM. Among the synthesized materials, maximum values of O/C and H/C (0.36 and 1.09) were expressed by HCK20 and HC, respectively. In any case, BC-based materials showed slightly decreased H/C values and increased O/C values compared to pristine BC. In contrast to pristine HC, HC-based materials showed slightly increased H/C values and decreased O/C values. The O/C ratio of BC (0.01), BCK10 (0.025), and BCK20 (0.02) was less than 0.2, suggesting more stability of such materials and a half-life of more than 1000 years^110^. In contrast, the O/C ratio of HC (0.360), HCK10 (0.331), and HCK20 (0.350) was between 0.2 and 0.6, suggesting a half-life ranging from 100 to 1000 years^110^. For BM, the O/C ratio was 0.604, which will probably possess a half-life of less than 100 years^110^). Likewise, low H/C values of BC and HC materials compared with BM indicated high aromaticity and reactivity^111^. In addition, the much lower H/C ratio of BC materials compared to HC materials demonstrated that BC materials were heavily carbonized and showed highly aromatic structures. Depending on IBI^112^ criteria, the H/C < 0.7 showed greater combined aromatic ring structures for the BC materials, while the H/C molar ratio of HC materials is higher than that of BC materials, which was ˃ 0.7. Therefore, BC materials showed higher aromaticity and low polarity than that of HC materials. Hence, the ratios of H/C and O/C in the BC-based materials and HC-based materials indicated more aromatic C and becoming less hydrophilic^113^.

#### TGA and recalcitrance index (R_50_)

**Figure 5:**
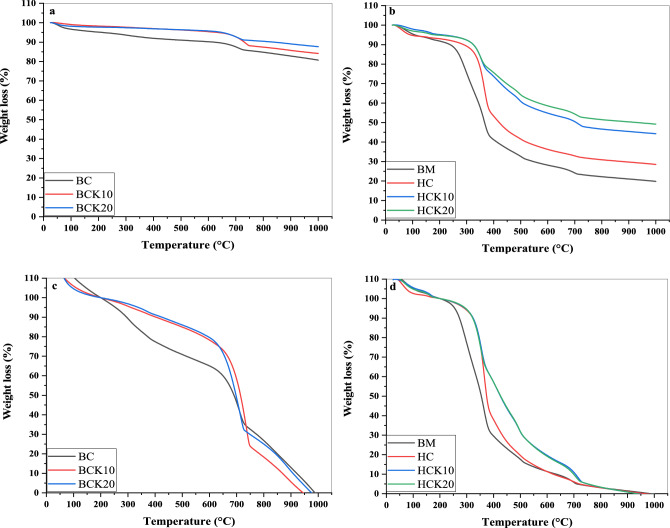
Thermogravimetric analysis (TGA) of (**a**) BC materials, (**b**) HC materials and BM, (**c**) moisture, ash and kaolinite corrected TGA thermograms of BC materials, (**d**) moisture, ash and kaolinite corrected TGA thermograms of HC materials and BM (Biochar (BC), BC with 10% kaolinite enrichment (BCK10), BC with 20% kaolinite enrichment (BCK20), Hydrochar (HC), HC with 10% kaolinite enrichment (HCK10), HC with 20% kaolinite enrichment (HCK20), BM: biomass).

In this study, TGA thermogravimetric analysis was applied to investigate the long-term stability of synthesized materials. The results of TGA-DTG analysis are shown in Fig. 5a,b. Our results indicated that the BM thermally decomposed earlier than the HC and BC materials due to thermal instability. The thermogravimetric curves of the different BC materials and the HC materials exposed similar performances as regards weight loss (%) on a reducing trend with rising temperature. For BC, BCK10, and BCK20, the sudden weight loss began at 650 °C ≈ 700 °C, at 350 °C ≈ 400 °C for HC, HCK10, and HCK20, and at 250 °C ≈ 350 °C for BM. The thermograms displayed two general regions where weight loss has occurred: (i) around 300°C for BM and HC materials because of thermal degradation of cellulose and hemicellulose compounds^114^ and (ii) around 600–1000 °C because of lignin degradation^51^. The order of materials for degradability is as follows: BCK20 < BCK10 < BC < HCK20 < HCK10 < HC < BM. Therefore, the BC and HC with a 20% kaolinite ratio were more stable, followed by a 10% kaolinite ratio, followed by pristine. Moreover, a higher weight loss was observed in HC materials Than in BC materials.

The recalcitrance of the synthesized materials in soil depends on the potential of BC and HC materials to resist thermal, physical, and chemical degradation. The formation of organometallic complexes and the aromatic carbon structure play significant roles in BC and HC materials. Due to its increased aromaticity, the C of BC materials has a higher potential for recalcitrance than that of HC materials and BM; however, the recalcitrance and stability of C in the soil after adding BC and HC materials vary depending on the kind and feedstock composition, the texture and structure of the soil, and other environmental factors. Hence, the recalcitrance index of BC and HC materials is required to be determined in relative to graphite, because one of the most stable C forms is graphite^115^. As a result, TGA thermograms that are marked as R_50_ were used by Harvey et al. ^47^ to create a novel recalcitrance index that predicts the materials' potential for recalcitrance.

To calculate the recalcitrant index (R_50_), TGA thermograms for all materials were corrected for moisture and ash contents, which reflect the stability of synthesized materials and the extent of C sequestration^116^. Figure 5c,d display moisture and ash-corrected TGA thermograms. Therefore, the R_50_ values of BM and HC in this study were 0.40 and 0.42, indicating that these are highly biodegradable and belong to class 3 (Table 3). BC, BCK10, and BCK20 were considered in class 1 with R_50_ of 0.78, 0.81, and 0.79, respectively, indicating that these have high recalcitrance potential^47^. The R_50_ values of HCK10 and HCK20 composites were 0.50 and 0.51, suggesting they were minimally degradable. Increased R_50_ in the BCK10 and BCK20 composites could be attributed to kaolinite's presence, which improved BC's oxidation resistance. The previous study by Ahmad et al.^51^ demonstrated that the higher R_50_ in silica BC composites could be related to the probable protection of C by silica particles, which may be controlled through the pyrolysis process. The reason was also attributed to the change from the aromatic C–C/C=C functionality to the C–O and C–H configuration of the BC surface forming stable organo-mineral complexes (i.e., C–O–Al and C–O–Si) with clay minerals^117^. The previous study by Wang et al.^115^ showed that the R_50_ increased by 0.89 for kaolinite-composited BC, reflecting that minerals-composited BC could improve the thermal stability of BC. According to Ahmad et al.^111^, changes in structural arrangements within the silica-BC complex may be responsible for the high recalcitrance and C sequestration potential of silica-composited BC. Therefore, increased R_50_ values of BC materials expect greater recalcitrance and stability because of the particular interactions of kaolinite with the BC matrix.

Although it provides a range of recalcitrance relevant to graphite (a highly stable C form), the recalcitrance index (R_50_) does not provide information on the specific timing for C sequestration. Consequently, the potential of C sequestration (CS) of BC and HC materials has been calculated using R_50_, yield, and C contents. A higher CS percentage relies on (i) the yield (%), (ii) the contents of C (%) before and after pyrolysis and hydrothermal carbonization, and (iii) the R_50_ value. In our study, the CS of the BC materials ranged from 46.43% to 51.63%, while the HC materials showed CS in the range of 29.16%–39.94%. The highest CS value was found in BCK20 (51.63), while the lowest was in HC. On the other hand, increased CS values were associated with an increasing percentage of kaolinite in BC and HC, which were 51.63% in BCK20 and 39.95% in HCK20 compared to pristine. Ahmad et al. ^111^ revealed that the silica presence in BC-based materials enhanced the C sequestration potential through modifications to the silica-BC complex's structural arrangements. Another study by Sewu et al. ^118^ indicated that the bentonite-BC materials had higher C sequestration potential than the original BC. Therefore, in our study, forming Si–O–C and Al–O–C in BC-based materials and HC-based materials possessed the highest C sequestration potential.

### Toxicity evaluation

#### Contents of PAHs

Biomass combustion is one of the primary sources of anthropogenic PAHs^119^. The total PAHs quantities and proportional contributions of individual PAHs provide valuable data regarding the quality of synthesized BC and HC materials regarding environmental safety. The contents of PAHs in BC and HC materials were determined to identify the PAHs retention in all materials due to various processes of pyrolysis and hydrothermal carbonization, and the findings are presented in Table 4. Pristine HC showed higher amounts of all PAHs than in pristine BC. Acenaphthene content was found to be around 2.6 times greater in pristine HC than pristine BC. Moreover, around 2 times greater phenanthrene, anthracene, and benzo [e] pyrene [BeP] contents, about 1.5 times greater naphthalene and acenaphthylene contents, and 1.3 times greater fluorine, fluoranthene, pyrene, and retene contents were detected in pristine HC in comparison to pristine BC. These findings support previous studies showing that pristine HC contains more PAHs than pristine BC^8,120^. Compared to dry pyrolysis, the tar condensation on the surface of HC through hydrothermal carbonization of biomass might have resulted in the retention of PAHs in such materials^58^. In contrast, the modification of BC and HC with kaolinite decreased the PAHs contents as compared to pristine BC and HC. The HC-based materials such as HCK10 and HCK20 showed a greater reduction of PAHs contents than the BC-based materials such as BCK10 and BCK20. Similarly, increasing kaolinite modification means more reduction of PAHs, such as in BCK20 and HCK20, which were more PAHs-reducing than BCK10 and HCK10. Generally, PAHs contents in HC-based materials were lower than in BC-based materials. Compared to pristine BC and HC, the reduction for these materials significantly ranged between 1.2 and 6.4 times for most of the PAHs contents. For instance, the reduction for phenanthrene was about 5 times for HCK10 and 6.4 times for BCK20 compared to HC. Similarly, the reduction of naphthalene was about 1.2 times for BCK10 and 2.6 times for BCK20 compared to pristine BC; likewise, about 2.8 times for HCK10 and 3.8 times for HCK20 as compared to pristine HC. Overall, ten forms of seventeen PAHs were detected in the BC and HC materials. The sum of the PAHs total concentrations of BC and HC materials ranged from 739.1 μg kg^−1^ in HCK20 to 2770.7 μg kg^−1^ in HC. The order of the ∑PAHs contents was HC (2770.7 μg kg^−1^) ˃ BC (1752.9 μg kg^−1^) ˃ BCK10 (1514.1 μg kg^−1^) ˃ HCK10 (972.8 μg kg^−1^) ˃ HCK20 (739.1 μg kg^−1^) ˃ BCK20 (934.4 μg kg^−1^). Nevertheless, ∑ 15 PAHs contents (∑16 US-EPA PAHs, except Benzo[b] Flouranthene) were in the BC (1487.8 μg kg^−1^), BCK10 (1293.3 μg kg^−1^), BCK20 (799.5 μg kg^−1^), HC (2383.2 μg kg^−1^), HCK10 (861.71 μg kg^−1^), and HCK20 (619.5 μg kg^−1^). Therefore, ∑15 PAHs contents in BC and HC materials were lower than the threshold value of ∑16 PAH (6000–20,000 μg kg^−1^ = 6000 μg kg^−1^) provided by the IBI^112^. Therefore, the PAHs compounds in BC and HC materials are considered safe for use as soil amendments and have the lowest potential risk for PAHs-related effects.

**Table 4:** The contents of polycyclic aromatic hydrocarbons (PAHs) (μg kg^-1^) in the synthesized materials.

No	PAHs	BC	BCK10	BCK20	HC	HCK10	HCK20
1	Naphthalene (Nap)	318.38	255.05	124.77	466.23	164.37	123.9
2	Acenaphthylene (Acy)	302.09	241.11	148.18	438.03	177.04	113.6
3	Acenaphthene (Ace)	118.36	153.67	107.69	308.55	103.22	96.45
4	Fluorene (Flu)	85.88	69.44	83.67	116.23	89.45	44.56
5	Phenanthrene (Phe)	205.56	205.41	119.45	398.49	77.87	62.33
6	Anthracene (Ant)	99.46	73.69	63.21	182.66	88.7	57.72
7	Fluoranthene (Fla)	152.43	109.45	57.38	198.34	77.51	49.44
8	Pyrene (Pyr)	205.67	185.44	95.19	274.67	83.55	71.51
9	Benzo[a] Anthracene (BaA)	ND	ND	ND	ND	ND	ND
10	Chrysene (Chr)	ND	ND	ND	ND	ND	ND
11	Benzo[k]Fluoranthene (BF)	ND	ND	ND	ND	ND	ND
12	Benzo[e] Pyrene (BeP)	42.55	38.21	31.29	89.35	27.23	24.31
13	Benzo[a] Pyrene (BaP)	ND	ND	ND	ND	ND	ND
14	Indeno[123-cd]Pyrene (InP)	ND	ND	ND	ND	ND	ND
15	Benzo [ghi] perylene (BghiP)	ND	ND	ND	ND	ND	ND
16	Dibenzo[ah] Anthracene (DBA)	ND	ND	ND	ND	ND	ND
17	Retene (Ret)	222.48	182.58	103.55	298.12	83.88	95.23
	∑ total PAHs	1752.9	1514.1	934.4	2770.7	972.82	739.1
	∑ 14 PAHs of 16 US-EPA PAHs	1487.8	1293.3	799.5	2383.2	861.71	619.5

Biochar (BC), BC with 10% kaolinite enrichment (BCK10), BC with 20% kaolinite enrichment (BCK20), Hydrochar (HC), HC with 10% kaolinite enrichment (HCK10), HC with 20% kaolinite enrichment (HCK20).

*ND* Non-detectable (below detection limit).

**Figure 6:**
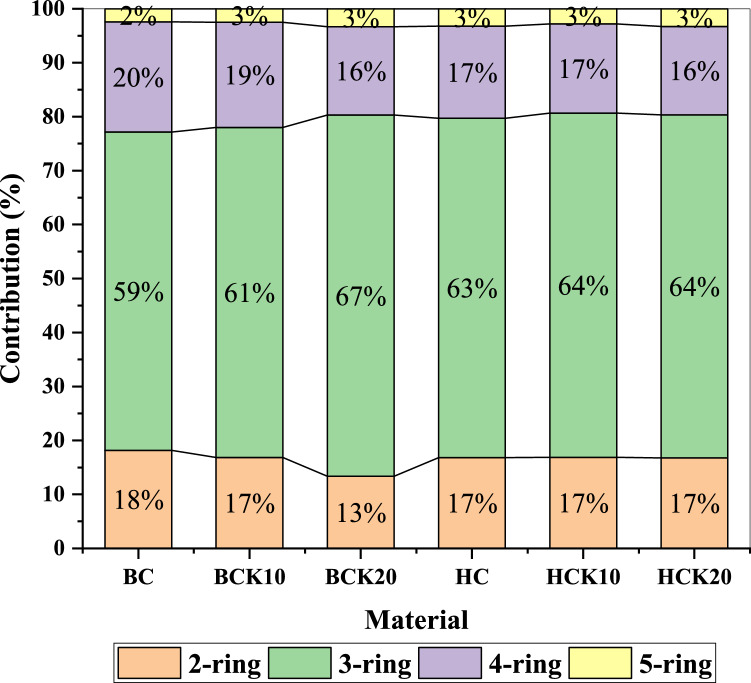
Percentage composition of 2-, 3-, 4-, 5-, and 6-rings PAHs in the BC and HC materials (2-ring PAHs include Naphthalene; 3-ring PAHs includes Acenaphthylene, Acenaphthene, Fluorene, Phenanthrene, Anthracene, and Retene; 4-ring PAHs includes Fluoranthene, Pyrene, Benzo[a] Anthracene, and Chrysene; 5-rings PAHs include Benzo[k]Fluoranthene, Benzo[a] Pyrene, and Benzo[e] Pyrene). Biochar (BC), BC with 10% kaolinite enrichment (BCK10), BC with 20% kaolinite enrichment (BCK20), Hydrochar (HC), HC with 10% kaolinite enrichment (HCK10), HC with 20% kaolinite enrichment (HCK20), BM: biomass.

Based on the aromatic rings number, PAHs with 3 rings were predominant in BC and HC materials, followed by those with 4, 2, and 5 rings (Fig. 6). Large proportion of low-molecular-weight 3 ring PAHs in BC and HC materials may be because of their atmospheric emission during pyrolysis and retention in the condensed tar through hydrothermal carbonization. Comparatively, pristine BC contained higher proportions of 2 and 4 ring PAHs than pristine HC, while pristine HC contained higher proportions of 3 and 5 ring PAHs. In BC and HC-based materials, the proportions of 2 and 5 ring PAHs were 17% and 3% in BCK10, BCK20, HCK10, and HCK20, which were equal (except 2 ring PAHs in BCK20 which, was 13%). The proportions of 3 ring PAHs were ordered as BCK20 (67%) ˃ HCK10 and HCK20 (64%) ˃ BCK10 (61%). Likewise, the proportions of 4 ring PAHs were ordered as BCK10 (19%) ˃ HCK10 and HCK20 (17%) ˃ BCK20 (16%). Generally, the proportion of 3 ring PAHs was the highest (ranging from 59 to 67%) in all synthesized materials, while the proportion of 5 ring PAHs was the lowest (ranging from 2 to 3%). During pyrolysis, they are released into the atmosphere while being retained in the condensed tar during hydrothermal carbonization. Low molecular weight 3 ring PAHs may be present in significant concentrations in HC. Previous studies mentioned that the toxicity and PAHs concentration in BC decreased significantly with increasing temperature, which can be attributed to the evaporation with rising temperature^121–123^. In addition, the most important factors in the elimination of PAHs compounds are volatility and thermal degradation^124^. Washing the BC and HC materials could reduce the negative impact on plant growth due to the reduced PAHs compounds^125^. Hence, kaolinite-composited BC and HC could be better options for amendments to soil and water due to decreasing PAHs contents.

Biochar (BC), BC with 10% kaolinite enrichment (BCK10), BC with 20% kaolinite enrichment (BCK20), Hydrochar (HC), HC with 10% kaolinite enrichment (HCK10), HC with 20% kaolinite enrichment (HCK20).

#### Maize germination and growth

**Figure 7:**
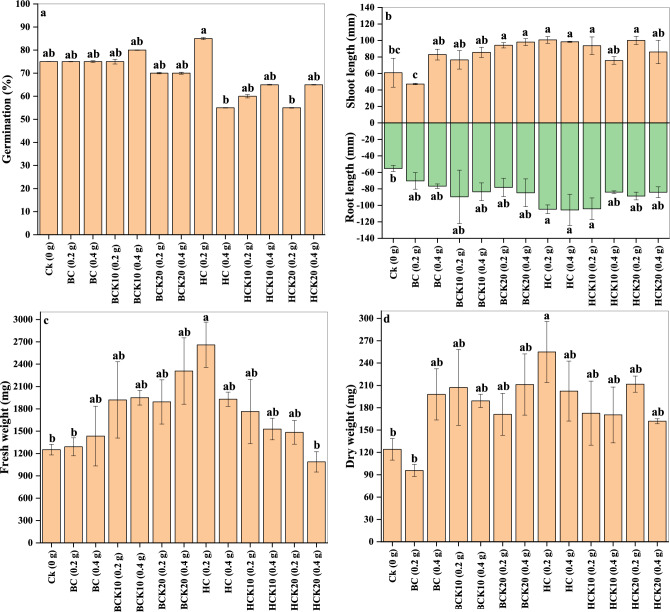
Germination test, shoot and root length, and fresh and dry weight of maize (*Zee mays* L.) affected by (Biochar (BC), BC with 10% kaolinite enrichment (BCK10), BC with 20% kaolinite enrichment (BCK20), Hydrochar (HC), HC with 10% kaolinite enrichment (HCK10), HC with 20% kaolinite enrichment (HCK20), BM: biomass).

The germination test results of maize (*Zea mays* L.) are presented in Fig. 7a. In control (CK) treatment, 75% germination of maize was detected. Seed germination was significantly affected by the application of BC and HC materials. The highest germination was observed in the HC (0.2 g) treatment, which was 85%, while the lowest was for HC (0.4 g) and HCK20 (0.2 g) treatments, which were 55%. In addition, there were no significant differences between 0.2 and 0.4 g applications of BC, BCK10, BCK20, and HCK10. The order of the germination percentages was as follows: HC (0.2 g) ˃ CK, BC (0.2 and 0.4 g), BCK10 (0.2 and 0.4 g), BCK20 (0.2 and 0.4 g), HCK10 (0.2 and 0.4 g), HCK20 (0.4 g) ˃ HC (0.4 g), HCK20 (0.2 g). Our finding showed that the previously mentioned synthesized materials are safe when applied to soil to enhance plant production without phytotoxic effects. Our finding supports the previous studies, which mentioned that the application of HC mixed with kaolinite could mitigate greenhouse gas emissions and might support improved soil retention of C and N for better management of agricultural nutrients^101^. Likewise, another study confirmed that applying a clay-BC composite positively impacted on the yield and quality of blue grass and improved soil properties^126^.

To evaluate the effect of various treatments, the shoot and root length of the maize seedling were also determined, and the results are shown in Fig. 7b. Compared to CK treatment, all treatments significantly improved the maize growth (except BC, 0.2 g). Shoot length of maize seedlings for BCK20 (0.2 and 0.4 g), HC (0.2 and 0.4 g), HCK10 (0.2 g), and HCK20 (0.2 g) treatments was 54.63%, 60.62%, 65.21%, 61.55%, 53.56%, and 64.20%, respectively, higher than CK. On the other hand, other treatments such as BC (0.4 g), BCK10 (0.2 and 0.4 g), HCK10 (0.4 g), and HCK20 (0.4 g) also increased the length of the shoot of maize seedlings by 36.11%, 25.62%, 40.60%, 24.36%, and 41.18%, respectively, as compared to CK. These findings confirm the potential of the synthesized materials to improve plant growth. Likewise, HC (0.2 and 0.4 g) and HCK10 (0.2 g) significantly increased the root length of maize seedlings by 89.27%, 90.80%, and 88.29%, respectively, compared to CK. Moreover, the root length of the other treatments was lower than previous treatments but higher than CK, which were 27.16% in BC (0.2 g), 38.71% in BC (0.4 g), 62.07% in BCK10 (0.2 g), 51.08% in BCK10 (0.4 g), 41.48% in BCK20 (0.2 g), 53.16% in BCK20 (0.4 g), 51.96% in HCK10 (0.4 g), 60.31% in HCK20 (0.2 g), and 52.06% in HCK20 (0.4 g).

Figure 7c,d depict maize seedlings’ fresh and dry weights as influenced by synthesized materials. The HC (0.2 g) treatment enhanced fresh and dry maize weights by 112.49% and 105.40%, respectively, compared to CK, while the other treatments were lower than HC (0.2 g) treatment. Hence, all the treatments significantly increased the fresh and dry weights, but in varying proportions compared to the CK treatment. Mumme et al.^127^ showed that biochar-zeolite composites increased the germination rate compared to the non-amended treatments. Another study by Medha et al.^41^ showed that Sorghum grass root and shoot length significantly increased than CK. Furthermore, among the tested materials, with the increase in the bentonite biochar composite and kaolinite fractions, the shoot and root length substantially increased by five and ten folds, respectively. In a study about the effect of biochar on the physiological growth of maize. Cong et al.^128^ found that biochar increased the dry biomass of maize by 22.22% against the control condition while up to 58.39% increase in plant height was noted against control with no biochar amendment. In another study, Yang et al.^129^ stated that kaolinite composite with walnut shell derived biochar improved its surface and adsorptive characteristics and nutrient release capacity, which ultimately positively impacted plant growth. Fregolente et al.^130^ found positive impact of hydrochar application on root and shoot development and dry biomass production of maize. Therefore, the parameters of seed germination and seedling growth revealed positive impacts of the synthesized materials with varying proportions. Consequently, it is highly recommended to properly assess the BC and HC materials before applying them to the soil as amendments.

The correlation between the characteristics of synthesized materials such as R_50_, CS, O/C, CEC, SA, and ∑ total PAHs toxicity and maize germination and growth were established (Table 5). The results of germination and growth parameters were taken by the average of additives rate. The relationship revealed a significant correlation between maize germination and growth and the characteristics of synthesized materials (R_50_, CS, O/C, CEC, SA, and PAHs). The significant relationships (r) between germination and characteristics of synthesized materials of R_50_, O/C, CEC, and SA were 0.75, − 0.78, − 0.78, and 0.81, respectively. Likewise, the r values between shoot length and R_50_, O/C, CEC, and SA were − 0.52, 0.54, 0.55, and − 0.71, respectively. Similarly, the r values between root length and R_50_, CS, O/C, CEC, and SA were − 0.78, − 0.83, 0.76, 0.78, and − 0.80, respectively. On the other hand, the r value between fresh weight and ∑ total PAHs was 0.53, while it was -0.50 between dry weight and SA. According to our findings, the characteristics of the synthesized materials have positive relationships on maize growth and germination.

**Table 5:** Relationship between the characteristics of synthesized materials and maize germination and growth.

Indices	R_50_	CS	O/C	CEC	SA	∑ PAHs	Germination	Shoot length	Root length	Fresh weight	Dry weight
R_50_	1.00										
CS	0.94*	1.00									
O/C	− 0.99*	− 0.92*	1.00								
CEC	− 0.99*	− 0.92*	1.00*	1.00							
SA	0.95*	0.83*	− 0.96*	− 0.97*	1.00						
PAHs	− 0.18	− 0.48	0.10	0.11	0.00	1.00					
Germination %	0.75*	0.49	− 0.78*	− 0.78*	0.81*	0.49	1.00				
Shoot length mm	− 0.52*	− 0.42	0.54*	0.55*	− 0.71*	0.03	− 0.46	1.00			
Root length mm	− 0.78*	− 0.83*	0.76*	0.78*	− 0.80*	0.48	− 0.31	0.67*	1.00		
Fresh weight mg	0.01	− 0.10	− 0.04	− 0.02	− 0.11	0.53*	0.36	0.58*	0.57*	1.00	
Dry weight mg	− 0.39	− 0.48	0.39	0.40	− 0.50*	0.48	0.00	0.82*	0.78*	0.78*	1.00

*Significant.

### Implications of the study

In recent years, fabricated BC and HC materials have received a lot of interest. Although a few studies have examined the feasibility of clay-supported BC and HC as composites in different applications, evidence from germination and growth parameters is still very limited. In our study, pristine BC, HC, and kaolinite-composited BC and HC were synthesized and characterized. The synthesized materials can be used successfully to sequester C, immobilize inorganic and organic pollutants in water and soil, increase N and phosphorus availability, and increase plant production at the field scale. Our founding materials, BC-based materials, could be used for long-term stability and sequestering C due to their high recalcitrance potential compared to HC-based materials, which had an R_50_ > 0.7 and CS > 47.63%. The higher stability and C sequestration of these charred materials could help mitigate climate change. Furthermore, synthesized materials can be used successfully for water and soil remediation; BC-based materials have high surface area and zeta potential, while HC materials have high CEC and surface functional groups. Moreover, kaolinite-composited BC and HC can be used for plant production, which showed no toxicity in the germination test and the lowest potential risk for PAHs-related impacts. On the other hand, HC-based materials typically have advantages such as a low pH and are suitable in arid regions that suffer from high pH; hence, their additions to the alkaline soil can overcome the alkalinity; as a result, the pH value decreases in these areas. In addition, the kaolinite-composited BC and HC materials can enhance soil health by improving the properties of soil through physical structure, increasing porosity, reducing bulk density, enhancing soil aggregation, organic contaminants degradation, water and nutrients retention, as well as climate change mitigation.

## Conclusion

Conocarpus waste-derived BC and HC were composited with kaolinite deposits and characterized for their chemical, proximate, elemental, and structural properties. Moreover, the potential toxicity of the synthesized materials was assessed for their implications as soil amendments. Analyses of the structural, morphological, and chemical properties revealed distinctions between pristine and designed BCs and HCs in terms of their properties. BC-based materials showed greater recalcitrance indices (R_50_: 0.79–0.81) and C sequestration potentials (47.63–51.63%) as compared to HC-based materials (R_50_: 0.50–0.51) and C sequestration (39.32–39.94). Kaolinite particles prevented C particles' thermal degradation, enhancing their stability. The O/C and H/C ratios were in the range of 0.02–0.025 and 0.15–0.15, respectively, in the BC-based materials, while 0.33–0.35 and 1.08–1.09, respectively, in HC-based materials, suggesting more aromaticity in BC-based materials as compared to HC-based materials. In addition, these materials can be utilized to sequester soil C pools for a longer time when used to amend the soil. BC-based materials showed a higher CEC than pristine BC, while HC-based materials showed a higher surface area than pristine HC. The total PAHs content decreased with increasing kaolinite deposits percentage in BC and HC; therefore, a greater reduction has been shown in BCK20 and HCK20 than in pristine chars. Overall, the total PAHs contents in BC and HC materials were below the USEPA's suggested limits. The removal of organic pollutants was mostly affected by the pyrolysis and hydrothermal carbonization processes; therefore, the thermal treatments can be a good way to generate chars with kaolinite deposits that have little or no organic pollutants and could be utilized as a safe soil amendment. Kaolinite-synthesized BC and HC demonstrated a positive impact on maize germination and seedling growth. In the future, kaolinite-synthesized BC and HC can be investigated for their efficacy as inexpensive soil amendments to improve soil health and plant productivity.

## Data Availability

The data analyzed during the current study are available from the corresponding author on reasonable request.
